# Supplementary material for: An interpretable deep learning model for detecting BRCA pathogenic variants of breast cancer from hematoxylin and eosin-stained pathological images
Source: PeerJ. 2024 Oct 28;12:e18098. doi: 10.7717/peerj.18098 (PMC11526788; doi:10.7717/peerj.18098)
Supplement: Supplemental Information 6 [file peerj-12-18098-s006.docx]

| BiAMIL | AUC  (95% CI) | Accuracy  (95% CI) | Sensitivity  (95% CI) | Specificity  (95% CI) | PPV  (95% CI) | NPV  (95% CI) | F1 score  (95% CI) |
| --- | --- | --- | --- | --- | --- | --- | --- |
| Fold 1 | 0.963  (0.896-1.000) | 0.889  (0.775-1.000) | 0.882  (0.765-0.999) | 0.895  (0.784-1.000) | 0.882  (0.765-0.999) | 0.852  (0.751-0.999) | 0.882  (0.765-0.999) |
| Fold 2 | 0.931  (0.838-1.000) | 0.829  (0.686-0.971) | 0.688  (0.508-0.867) | 0.947  (0.866-1.000) | 0.917  (0.815-1.000) | 0.782  (0.625-0.940) | 0.786  (0.629-0.943) |
| Fold 3 | 0.918  (0.816-1.000) | 0.857  (0.726-0.989) | 0.813  (0.664-0.961) | 0.895  (0.781-1.000) | 0.867  (0.739-0.994) | 0.850  (0.716-0.984) | 0.839  (0.699-0.978) |
| Fold 4 | 0.819  (0.672-0.966) | 0.823  (0.678-0.969) | 0.812  (0.663-0.962) | 0.833  (0.691-0.975) | 0.812  (0.663-0.962) | 0.833  (0.691-0.975) | 0.812  (0.663-0.962) |
| Fold 5 | 0.976  (0.920-1.000) | 0.853  (0.719-0.987) | 0.875  (0.751-0.999) | 0.833  (0.691-0.975) | 0.823  (0.678-0.969) | 0.882  (0.761-1.000) | 0.848  (0.712-0.984) |
