# Supplementary material for: An interpretable deep learning model for detecting BRCA pathogenic variants of breast cancer from hematoxylin and eosin-stained pathological images
Source: PeerJ. 2024 Oct 28;12:e18098. doi: 10.7717/peerj.18098 (PMC11526788; doi:10.7717/peerj.18098)
Supplement: Supplemental Information 7 [file peerj-12-18098-s007.docx]

| ResNet 34 | AUC  (95% CI) | Accuracy  (95% CI) | Sensitivity  (95% CI) | Specificity  (95% CI) | PPV  (95% CI) | NPV  (95% CI) | F1 score  (95% CI) |
| --- | --- | --- | --- | --- | --- | --- | --- |
| Fold 1 | 0.889  (0.774-1.000) | 0.861  (0.734 -0.988) | 0.882  (0.765-0.999) | 0.842  (0.708-0.976) | 0.833  (0.696-0.971) | 0.889  (0.775-1.000) | 0.857  (0.729-0.985) |
| Fold 2 | 0.888  (0.771-1.000) | 0.828  (0.686-0.971) | 0.812  (0.664-0.961) | 0.842  (0.704-0.980) | 0.812  (0.664-0.961) | 0.842  (0.704-0.980) | 0.812  (0.664-0.961) |
| Fold 3 | 0.838  (0.700-0.978) | 0.800  (0.647-0.952) | 0.875  (0.751-0.999) | 0.737  (0.567-0.906) | 0.737  (0.567-0.906) | 0.875  (0.751-0.999) | 0.800  (0.647-0.952) |
| Fold 4 | 0.840  (0.701-0.979) | 0.823  (0.678-0.969) | 0.812  (0.663-0.962) | 0.833  (0.691-0.975) | 0.812  (0.663-0.962) | 0.833  (0.691-0.975) | 0.812  (0.663-0.962) |
| Fold 5 | 0.892  (0.776-1.000) | 0.794  (0.639-0.950) | 0.812  (0.663-0.962) | 0.778  (0.617-0.938) | 0.765  (0.600-0.928) | 0.823  (0.680-0.969) | 0.788  (0.630-0.945) |
