# Supplementary figures and images for: An interpretable deep learning model for detecting BRCA pathogenic variants of breast cancer from hematoxylin and eosin-stained pathological images
Source: PeerJ. 2024 Oct 28;12:e18098. doi: 10.7717/peerj.18098 (PMC11526788; doi:10.7717/peerj.18098)

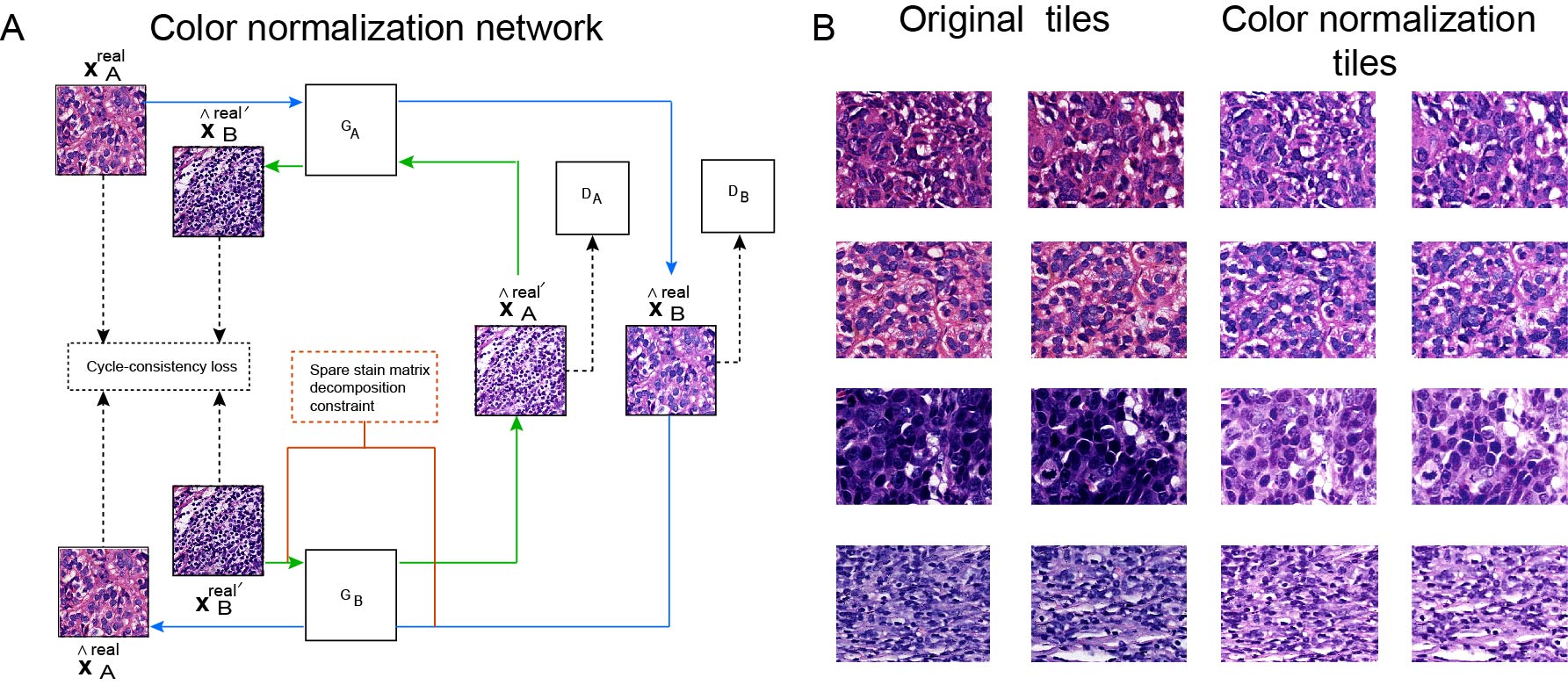

Supplement: Supplemental Information 9 — (A). Workflow of sparse stain matrix decomposition constraint Cycle GAN color normalization model (SDCC-GAN). (B). Representative tiles of the color normalization network. The original tiles are in the first and second rows; The corresponding color-normalized tiles are in the third and fourth rows. [file peerj-12-18098-s009.jpg]

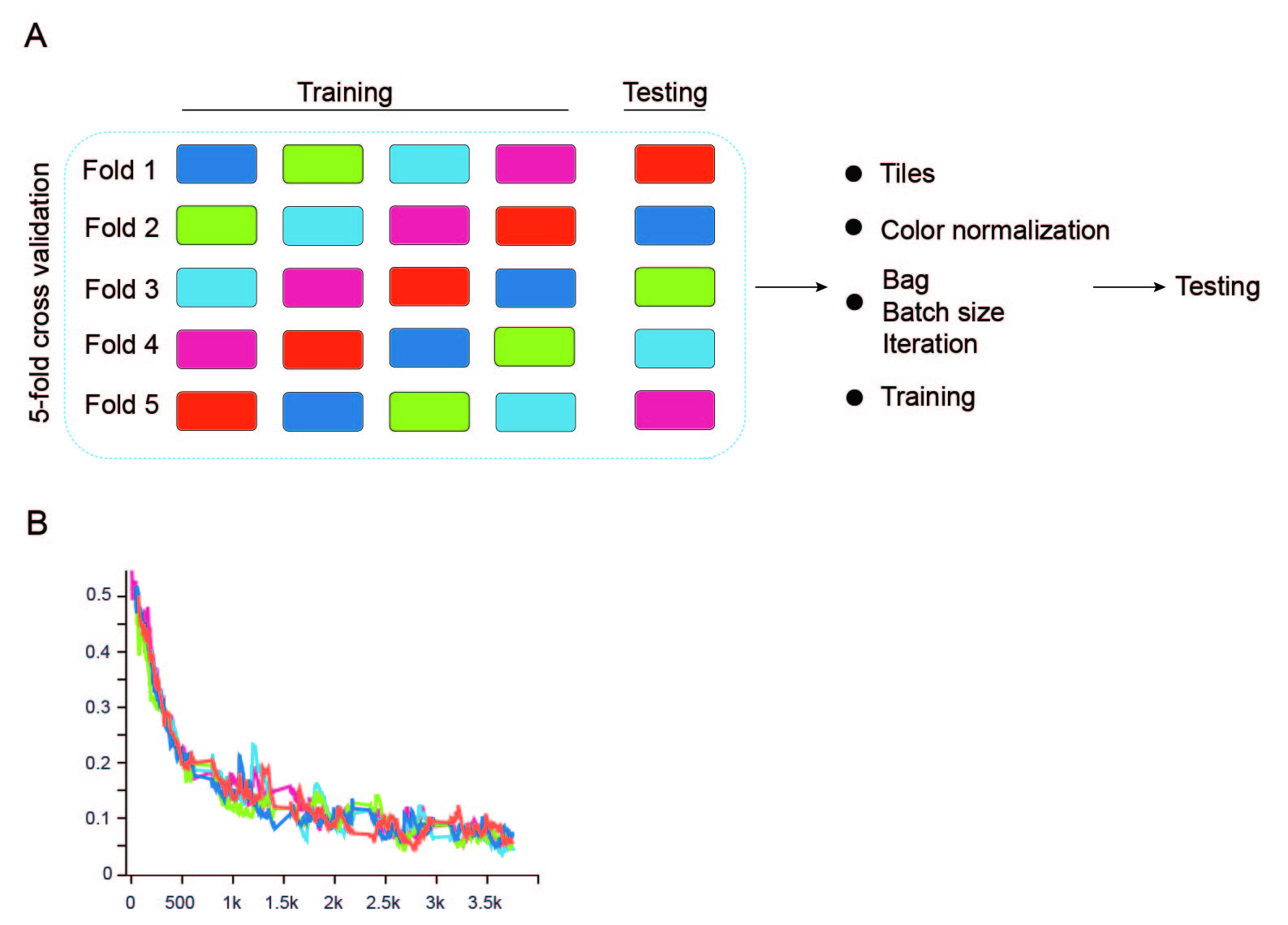

Supplement: Supplemental Information 10 [file peerj-12-18098-s010.jpg]
